# Management and research efforts are failing dolphins, porpoises, and other toothed whales

**DOI:** 10.1038/s41598-024-57811-7

**Published:** 2024-03-25

**Authors:** Andrew J. Temple, Ute Langner, Michael L. Berumen

**Affiliations:** Red Sea Research Center, King Abdullah University of Science and Technology, Thuwal, Saudi Arabia

**Keywords:** Marine mammal, Cetacean, Fisheries, Habitat degradation, Extinction, Conservation, Biodiversity, Conservation biology

## Abstract

Despite being subject to intensive research and public interest many populations of dolphins, porpoises, and other toothed whales (small cetaceans) continue to decline, and several species are on the verge of extinction. We examine small cetacean status, human activities driving extinction risk, and whether research efforts are addressing priority threats. We estimate that 22% of small cetaceans are threatened with extinction, with little signs of improvement in nearly thirty years. Fisheries and coastal habitat degradation are the main predictors of extinction risk. Contrary to popular belief, we show that the causal impact of small-scale fisheries on extinction risk is greater than from large-scale fisheries. Fisheries management strength had little influence on extinction risk, suggesting that the implementation of existing measures have been largely ineffective. Alarmingly, we find research efforts for priority threats to be vastly underrepresented and so a major shift in research focus is required. Small cetaceans are among the lower hanging fruits of marine conservation; continued failure to halt their decline bodes poorly for tackling marine biodiversity loss and avoiding an Anthropocene mass extinction.

## Introduction

Charismatic marine vertebrates have been the focus of more intensive research, public interest, and management effort than their other vertebrate and invertebrate counterparts. The heightened interest in species like sharks, marine mammals, and sea turtles is in part due to their charismatic status, but they are also economically valuable for fisheries and tourism^1,2^; they influence the structure, dynamics, and function of ecosystems^3–5^; promote connectivity among ecosystems through nutrient cycling^6,7^; and can be used as sentinel species to monitor ocean health^8,9^ in a time of unprecedented environmental change. Despite this, charismatic marine vertebrates are not protected from the ongoing biodiversity loss in the world’s oceans, with many taxa, species, and populations increasingly at risk of extinction^10–12^. Clearly, being subject to higher levels of scientific and public interest alone is not enough to protect species from extinction. If we cannot halt the decline of charismatic marine vertebrates, what hope is there for those taxa which receive far less attention?

Among the most charismatic and researched marine vertebrate groups are the small cetaceans. While the definition of ‘small cetacean’ is not universally agreed, we here consider them to be the dolphins, porpoises, and all other odontocetes (superfamily Odontoceti) except for the sperm whale *Physeter macrocephalus*. Unlike other marine mammals, such as the great whales (the sperm whale and the baleen whales, superfamily Mysticeti) and pinnipeds (seals, sea lions, and walrus, families Odobenidae, Otariidae, and Phocidae), small cetaceans did not experience severe declines from historic industrialised commercial overharvesting^13,14^. Nor, like the chondrichthyans (sharks, rays, and chimaeras, parvphylum Chondrichthyes) and the bony fish (parvphylum Osteichthyes), are small cetaceans subject to ongoing and widespread commercial harvest. Yet the Baiji (*Lipotes vexillifer*) is already likely to be extinct^15^ and the Vaquita (*Phocoena sinus*), the Atlantic humpback dolphin (*Sousa teuszii*), and populations of other several species around the world sit on the brink of extinction^11,16^. What then, is driving extinction risk in small cetaceans?

Unintentional catches, or bycatch, in fisheries are widely considered to be the main driver of extinction risk in small cetaceans^11,17–19^. Annual catches in large-scale, industrialised fisheries alone are likely to be in the hundreds of thousands of individuals per year^17,20^ and though catches in small-scale fisheries are largely unknown they may be similar in scale^19,20^. Other threats are also increasingly recognised. For example, persistent organic pollutants are responsible for reduced reproductive fitness^21^, plastics and marine litter have caused or contributed to deaths of a growing number of individuals^22^, and anthropogenic noise can result in acute and chronic impacts like barotrauma, displacement, and reduced fitness^23^. Beyond these immediate threats there is also the looming threat from climate change^24^. Small cetacean populations are susceptible to even low levels of non-natural mortalities because of their slow growth, late maturity, and low reproductive rates^25–27^. If we are to halt biodiversity loss in small cetaceans, or indeed in any taxonomic group, then we must understand not only what threats species face, but also the relative importance of those threats. We must then tailor research and management efforts accordingly, if we are to have any meaningful chance of success.

To this end, here we use the International Union for the Conservation of Nature (IUCN) Red List of Threatened Species (hereafter “IUCN Red List”) and the current peer-reviewed literature to answer the following key questions: (a) what are the historic and current levels of extinction risk faced by small cetaceans? (b) what are the key drivers of extinction risk in small cetaceans and how important are they? and, (c) do priority threats receive appropriate levels of research effort from the scientific community?

## Results

### No improvement in threatened status for 30 years

**Figure 1:**
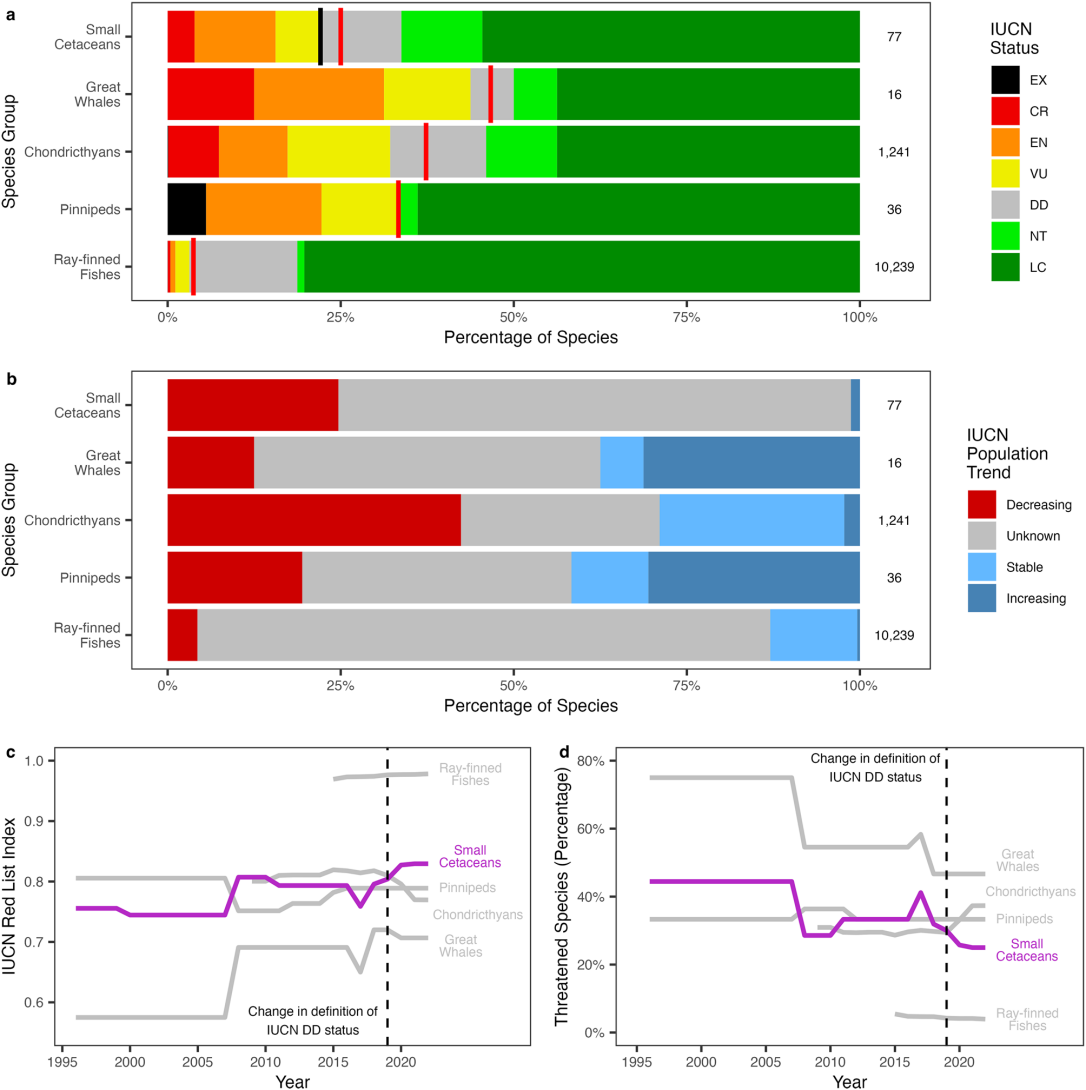
Red List status and trends for large marine vertebrate groups. (**a**) IUCN Red List status of small cetaceans and other large marine vertebrate groups, red vertical lines indicate the best estimate of threat status under the assumption that the status of Data Deficient (DD) species is proportional to data-sufficient species, black vertical line indicates the estimated threat status when Data Deficient small cetacean species status’ are estimated in this study. (**b**) IUCN Red List population trends of small cetaceans and other large marine vertebrate groups. (**c**) Trends in the IUCN Red List Index of selected marine vertebrate groups. (**d**) Trends in the threatened (Extinct (EX), Critically Endangered (CR), Endangered (EN), or Vulnerable (VU)) status of selected marine vertebrate groups. Other status classes are Near Threatened (NT), and Least Concern (LC). For (**a**) and (**b**) number of species is indicated on the right of each bar.

To assess and contextualise the threatened status of marine small cetaceans, we used data from the IUCN Red List^16,28^. Using an estimate based on the current distribution of IUCN Red List assessments (*n* = 77) as much as one-quarter (25.0%) of small cetaceans may be threatened with extinction (Critically Endangered, Endangered, or Vulnerable). Of species with sufficient data to be assessed (*n* = 68) 4.41% (*n* = 3) are Critically Endangered, 13.2% (*n* = 9) are Endangered, 7.35% (*n* = 5) are Vulnerable, 13.2% (*n* = 9) are Near Threatened, and 61.8% (*n* = 42) are Least Concern. Nine species are currently Data Deficient. Small cetaceans are less threatened than other marine vertebrate groups which display similar life-history characteristics, such as the great whales (46.7%), pinnipeds (33.3%), and chondrichthyans (37.3%), but are more threatened than marine bony fish (3.73%). At present only 20 species have had their population trends assessed, 19 of which are believed to have decreasing populations, no species have yet been assessed to have stable populations, and only 1 species has been assessed as having an increasing population^16,28^. Further, the proportion of species threatened with extinction and IUCN Red List Index^16^ (the mean across species groups when Extinct species are assigned a value of 0.0, Critically Endangered a value of 0.2, Endangered 0.4, Vulnerable 0.6, Near Threatened 0.8, and Least Concern 1.0) of small cetaceans has remained roughly stable for nearly 30 years. Fluctuations in the Red List Index are mainly in response to the discovery of new species or the re-assessment of previously Data Deficient species following new guidelines on the assignment of Red List Status to previously Data Deficient species (Fig. 1), which mask an overall decline in small cetacean Red List Status^28^. Taken together, this suggests that small cetaceans are likely to be experiencing a continued but gradual decline worldwide.

### Data only partially support IUCN ranking of threats

**Figure 2:**
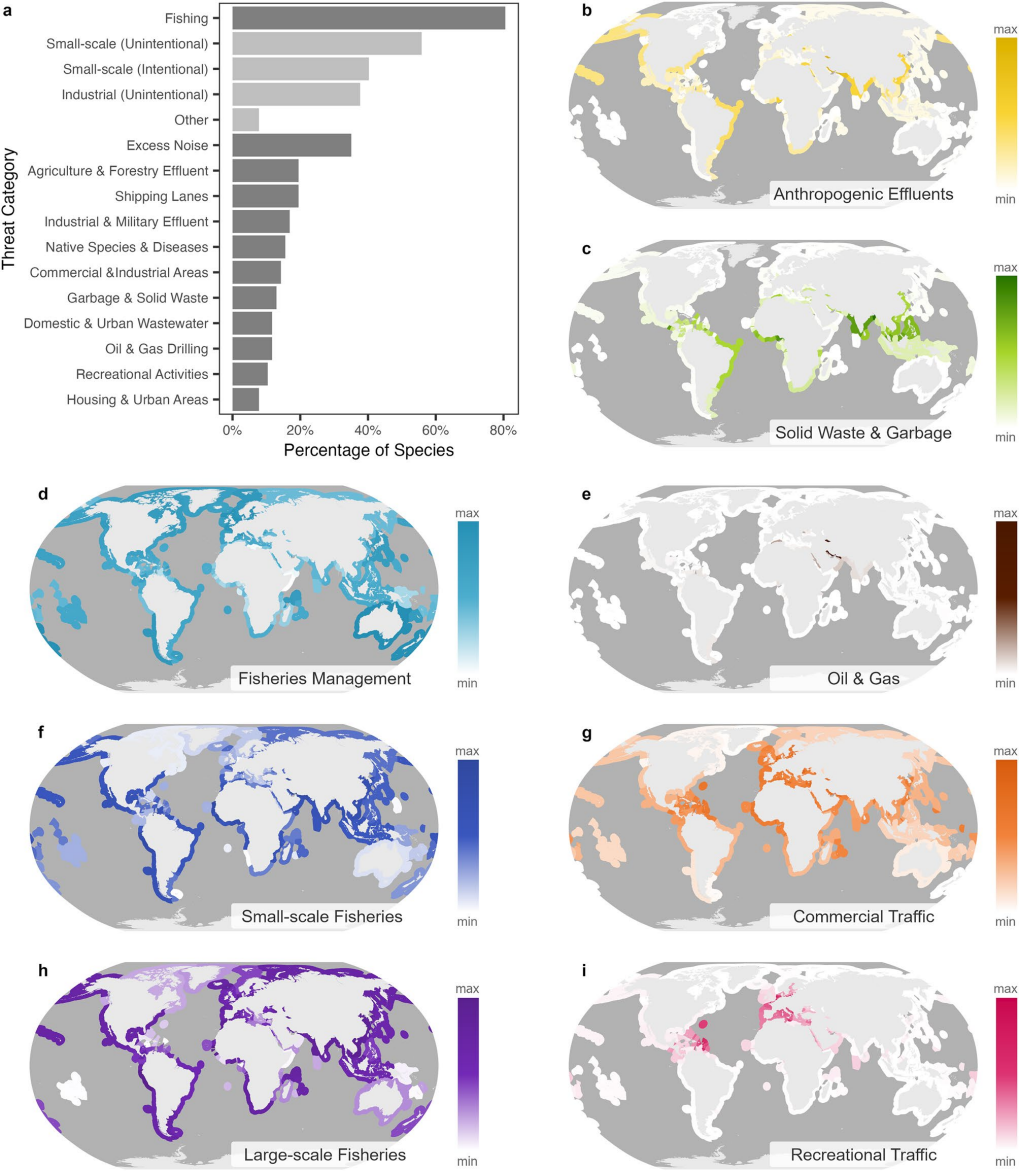
Threats to small cetaceans and the distribution of threat proxies used in this study. (**a**) Threats attributed as having “High” or “Medium” impacts on small cetaceans in IUCN Red List assessments. Relative levels of threat proxies per unit of coastline length by country for (**b**) anthropogenic effluents, (**c**) solid waste and garbage, (**d**) fisheries management, (**e**) oil and gas, (**f**) small-scale fisheries pressure, (**g**) commercial and passenger vessel traffic, (**h**) large-scale fisheries, and (**i**) recreational vessel traffic. Variables displayed in (**b**–**i**) have been log transformed to improve their distribution. See Methods for details on data sources for (**b**–**i**).

Threats to small cetaceans listed by the IUCN^16^ can be broadly categorised into three groups: fisheries, other human activities in the marine environment, and habitat degradation. Fisheries are the most attributed threat to small cetaceans, being listed as either “Medium” or “High” impact levels to 80.5% of all species, and are the only threat attributed as “High” impact to any species. Unintentional (55.8% of species) and intentional (40.3% of species) catches in small-scale fisheries are attributed as threats more commonly than unintentional threats from the large-scale industrial fleets (37.7% of species). Impacts from other human activities in the marine environment include anthropogenic (or “Excess”) noise, which is attributed as having “Medium” impacts on 35.1% of small cetaceans, shipping lanes and the associated risk of ship-strike, and oil and gas drilling activities. Various causes of habitat degradation are also commonly listed, such as agricultural, forestry, industry, military, and urban effluents and wastewater; garbage and solid waste discharge into the marine environment; and urbanisation and development along the coastline (Fig. 2).

**Figure 3:**
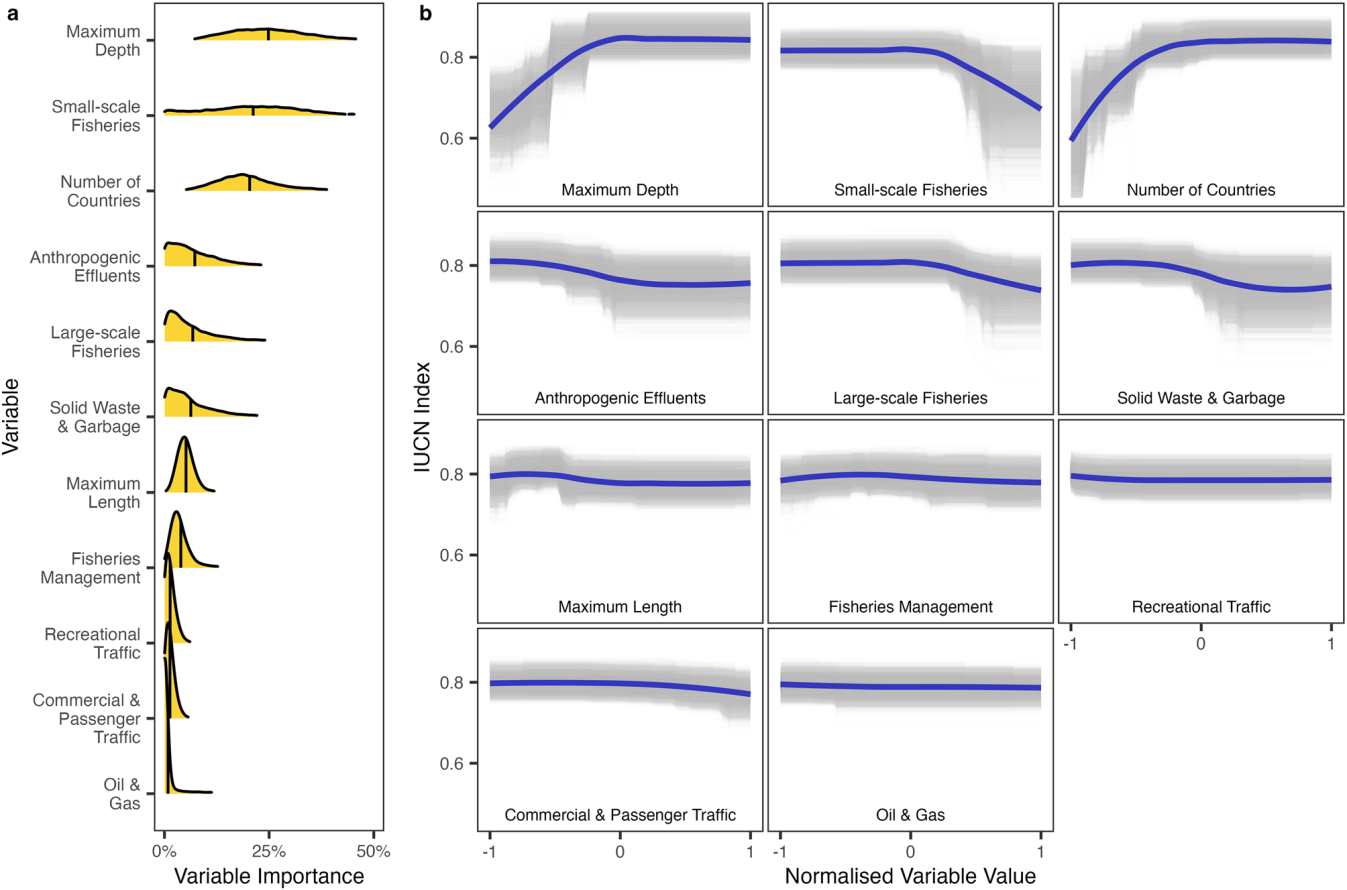
Relationships between IUCN Red List Index and key threats. (**a**) Variable importance for predicting IUCN Red List Index, the mean variable importance is indicated with a vertical black line. (**b**) Partial dependence plots showing relationships between variables and IUCN Red List Index, grey lines represent each of the 10,000 bootstrap iterations of the model, purple lines represent average trends.

We analysed the major threats outlined by the IUCN and their capability to predict the extinction risk of small cetaceans, alongside variables expected to mediate species’ exposure to threats (Fig. 3). The model was able to distinguish well between threatened (IUCN Red List Index < 0.7) and non-threatened (IUCN Red List Index > 0.7) species, with a balanced accuracy of 83.6% across all iterations. Finer-scale predictive accuracy was variable particularly when distinguishing among threatened categories, with balanced accuracies of 82.6% (Least Concern), 57.1% (Near Threatened), 64.9% (Vulnerable), 56.3% (Endangered), and 58.9% (Critically Endangered). Our results partially align with the IUCN, showing fisheries to be the greatest predictor of small cetacean threatened status, but suggest that the perceived importance of other threats may need to be reconsidered.

### Fishing pressure, habitat degradation, and species’ exposure predict extinction risk

Fisheries had the strongest predictive power of the threat groups considered with a combined average variable importance (AVI) of 32.1%. IUCN Red List Index declined with increasing small- and large-scale fishing pressure, and only improved very slightly with increasing fisheries management. Small-scale fishing pressure (21.4% AVI) was by far the largest contributor, more than three times greater than large-scale fishing pressure (6.78% AVI), with fisheries management (3.91% AVI) providing the lowest predictive power of fisheries related variables. The relative difference between the predictive power of small- and large-scale fishing pressure suggests that small-scale fisheries may contribute far more to the extinction risk of small cetaceans. The low relative importance of fisheries management suggests that countries with high governance strength are failing to improve the overall conservation of small cetacean species found in their waters.

Habitat degradation was the second most important threat group, with a combined AVI of 13.6%. Increasing levels of anthropogenic effluent inputs (7.26% AVI) were a stronger predictor than increasing solid waste and garbage inputs (6.31% AVI), with both leading to a decline in IUCN Red List Index. The importance of anthropogenic effluents and solid waste and garbage as predictors likely reflects the degradation of habitats associated with coastal development, urbanisation, and the inputs of marine litter and pollutants into coastal ecosystems. Other human activities in the marine environment were relatively unimportant compared to fisheries pressure and habitat degradation, with a combined AVI of 3.54%. Recreational vessel traffic (1.36% AVI), commercial and passenger vessel traffic (1.31% AVI), and oil and gas (0.867% AVI) were weak predictors of small cetacean extinction risk.

The strongest predictors of extinction risk were traits which mediate the exposure of species to human impacts. IUCN Red List Index markedly increased with increasing maximum water depths (25.2% AVI) and increases in the number of countries across which species were distributed (20.4% AVI). Given the limited impact of fisheries management on IUCN Red List Index, the predictive power of number of countries may indicate that widely distributed species benefit from more natural refuges from anthropogenic impacts and have larger overall abundances making them more resilient to spatially heterogeneous threats like fisheries pressure. Similarly, species found over increasing maximum water depths are less likely to be subject to intensive anthropogenic pressures. Maximum size (5.15% AVI) was a weaker predictor of extinction risk.

### Data deficient species are unlikely to be threatened with extinction

**Figure 4:**
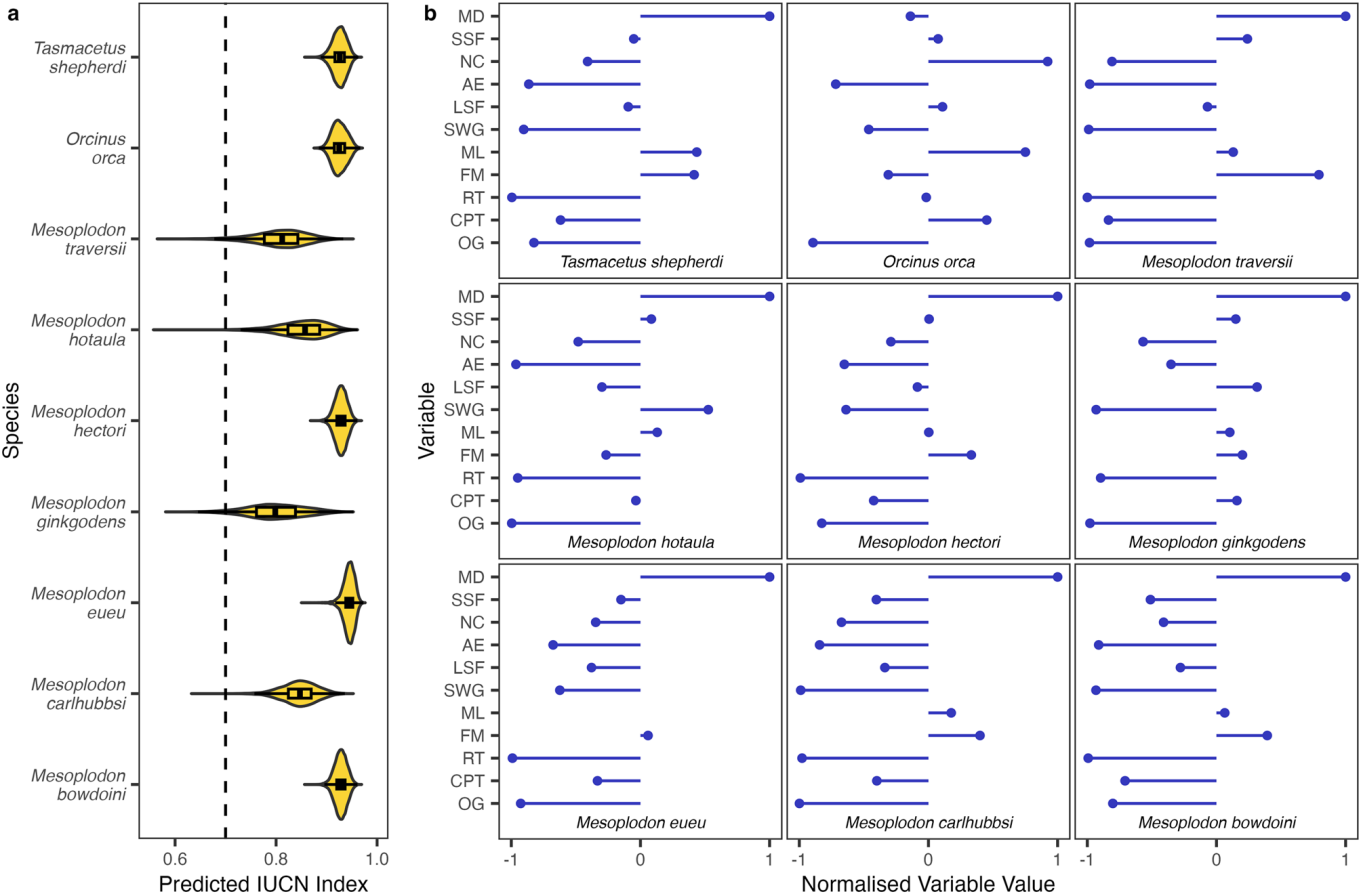
Predicted IUCN Red List index for Data Deficient Species. (**a**) Distribution of IUCN Red List index predictions from 10,000 bootstrap iterations of the model. (**b**) Predictor variables values for each species. Number of countries (NC), maximum depth (MD), small-scale fisheries (SSF), solid waste and garbage (SWG), anthropogenic effluents (AE), large-scale fisheries (LSF), fisheries management (FM), maximum length (ML), oil and gas (OG), recreational traffic (RT), and commercial and passenger traffic (CPT).

The models were then used to predict the IUCN Red List Index for the nine Data Deficient small cetacean species (Fig. 4), *Mesoplodon bowdoini*, *Mesoplodon carihubbsi*, *Mesoplodon eueu*, *Mesoplodon ginkgodens*, *Mesoplodon hectori*, *Mesoplodon hotaula*, *Mesoplodon traversii*, *Orcinus orca*, and *Tasmacetus shepherdi*. Understanding of the distributions of the Data Deficient beaked whales is generally poor, the predictions presented here are based on the best currently available data for these species and should therefore be revisited as our understanding of beaked whale distributions improve. Apart from *Orcinus orca*, all currently Data Deficient species are beaked whales, which appear to be strictly oceanic and are found across deep waters away from the most intensive human impacts. Conversely, *Orcinus orca* is among the most widely distributed of all animal species. There are several ecotypes of *Orcinus orca* and it may in fact represent a species complex^29^, here we assess it as a single species but this assessment should be revisited if multiple species are formally recognized in future. The mean predicted IUCN Red List Index for all Data Deficient species was greater than 0.7, indicating that these species are most likely Near Threatened or Least Concern, and suggesting that recent estimates which speculated that one or two of these species are likely to be threatened may be overstated^28^.

### Small-scale fisheries drive extinction risk more than large-scale fisheries

**Figure 5:**
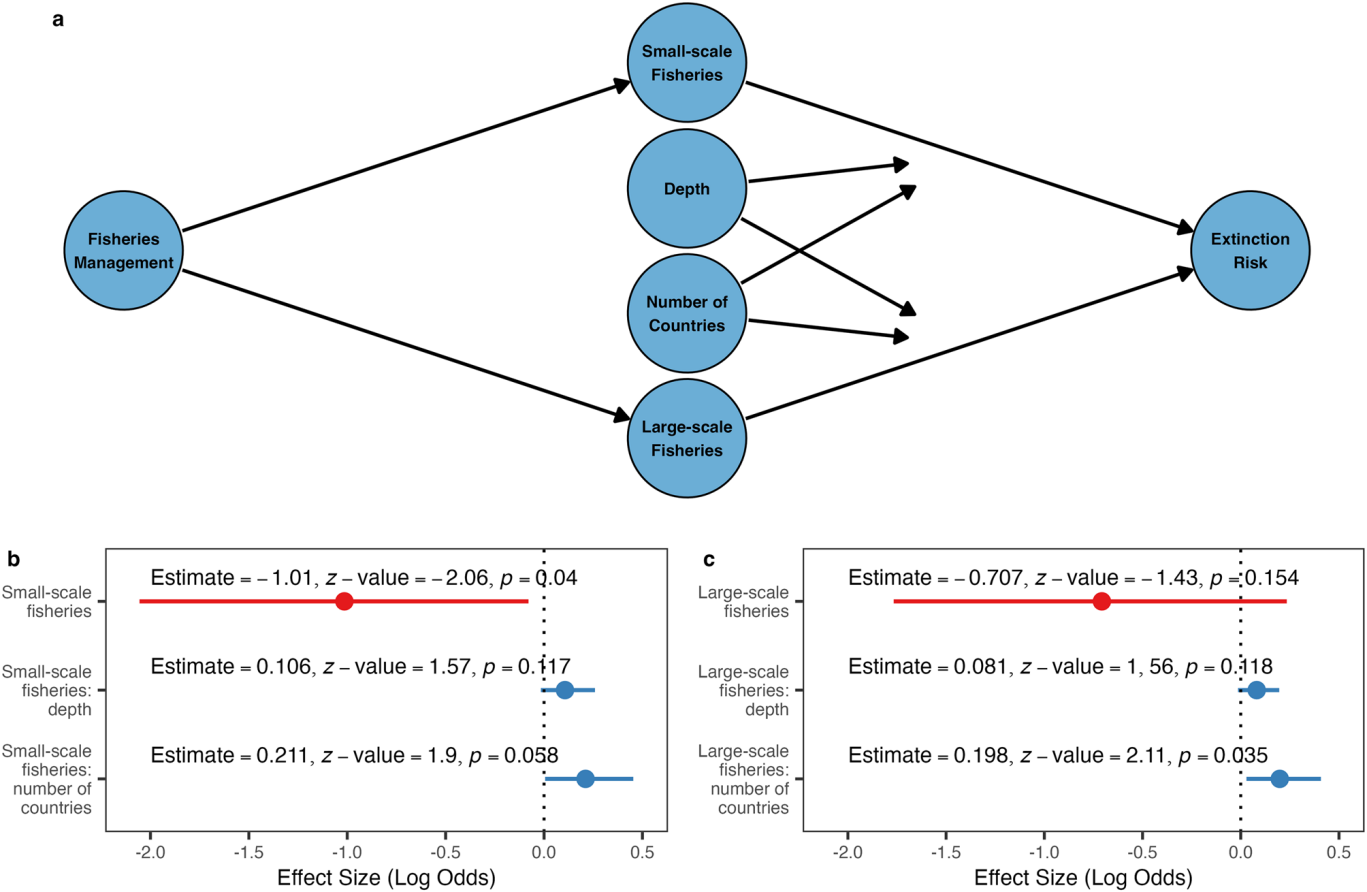
Causal effects of small- and large-scale fisheries on extinction risk of small cetaceans. (**a**) Directed Acyclic Graph outlining the proposed causal structure for fisheries impacts on extinction risk, fisheries management exert control on small- and large-scale fishing pressure, species maximum water depth of species distribution and the number of countries they are distributed across moderate fishing pressure impact on extinction risk. Model outputs estimating causal impact of fisheries on extinction risk and mediating effects for (**b**) small-scale fisheries and (**c**) large-scale fisheries.

The predictive model suggests that small-scale fisheries are a stronger predictor of increasing extinction risk than their large-scale counterparts. We also found more evidence to suggest that increasing pressure from small-scale fisheries has a stronger causal effect on increasing extinction risk than large-scale fisheries do. In the case of both large- and small-scale fisheries we also found evidence to suggest that increasing maximum depth and number of countries reduce the causal impact of fisheries pressure on extinction risk (Fig. 5). We found no evidence for any effect of increasing fisheries management on the extinction risk of species (small-scale fisheries, *z*-value = 0.570, *p* = 0.569; large-scale fisheries, *z*-value = 0.888, *p* = 0.374). Our finding suggests that small-scale fisheries have a greater overall impact on the extinction risk of small cetaceans and should be considered a higher priority threat, this corresponds well with the relative threat rankings of small- and large-scale fisheries on the IUCN Red List (Fig. 2).

### Research effort does not match with probable threat priorities

**Figure 6:**
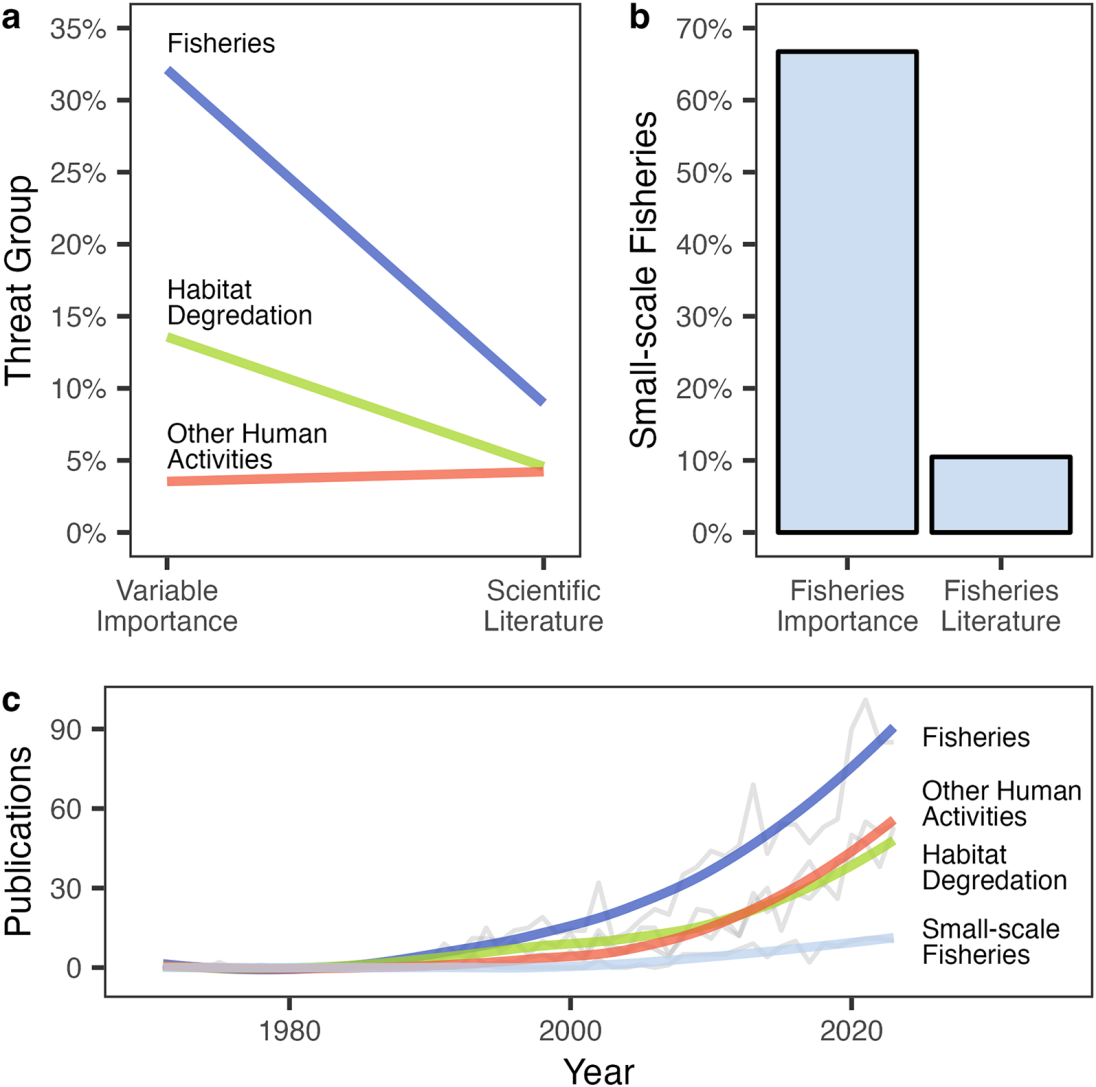
The focus of small cetacean research efforts and the predictive importance of threats. (**a**) The combined average variable importance (AVI) of threat groups in predicting small cetacean extinction risk as identified in this study, compared to the proportion of the peer-reviewed scientific literature which addressed these threats. (**b**) The contribution of small-scale fisheries to the combined AVI of fisheries and to the fisheries scientific literature. (**c**) Annual number and the smoothed averages of publications addressing each threat group by year from 1970 to 2023.

The predictive power of anthropogenic pressures are likely to be reflective of their relative impacts because all share an explicit causal link with extinction risk. Fisheries result in direct mortality, habitat degradation may reduce the size of populations which ecosystems can support, and noise-based disturbances may result in reduces animal fitness. Having established the relative importance of different threats in predicting the extinction risk of small cetaceans, and providing evidence that at least for fisheries the causal effect of small-scale fisheries pressure is greater than that of large-scale fisheries, we then compared these to the proportion of scientific literature that addressed these threats, taken from a sample of 13,507 publications (Fig. 6). We find that the proportion of the small cetacean scientific literature which addresses fisheries (8.97%) and habitat degradation (4.57%) topics is far below that of their relative AVIs (32.1% and 13.6%, respectively), whereas the proportion of literature addressing the impacts of other human activities in the marine environment (4.21%) was slightly above its AVI (3.54%). The differences in AVI and proportion of the scientific literature suggest that research effort in both fisheries and habitat degradation are substantially underrepresented despite their importance. Further, the annual level of research outputs on the impacts of other human activities is now roughly equal to that of habitat degradation and looks likely to exceed it in the coming years. A closer inspection of the fisheries literature in particular highlights an alarming lack of research relating to small-scale fisheries. Small-scale fisheries were the most important threat predictor of extinction risk (21.4% AVI), accounting for 66.7% of combined fisheries AVI. Despite this, small-scale fisheries related research only accounts for 0.940% of all small cetacean research and only 10.5% of all fisheries related research in small cetaceans.

## Discussion

Small cetaceans have shown little signs of improvement in nearly 30 years, despite being subject to more intensive research and widespread public attention than most other marine taxa. We estimate that 22.1% of small cetacean species are threatened with extinction and show that fisheries, particularly small-scale fisheries, and habitat degradation are the key threats which predict extinction risk. Other commonly cited threats, such as environmental noise and oil and gas exploration^16^, play only a minor role in predicting extinction risk and are likely restricted to localised impacts only. We also provide direct evidence to suggest that the causal effect of small-scale fisheries on the overall extinction risk of small cetaceans is greater than that of their large-scale counterparts. Both predictive and causal analyses give further evidence to support previous works showing that extinction risk is highest for those species restricted to shallower coastal areas and with smaller geographic ranges^11,12,28^ because these result in an increased relative exposure to human impacts and result in reduced natural refuges. Our findings suggest that research, conservation, and management efforts have so far been largely unsuccessful in reversing the decline of small cetaceans at the global scale. This failure bodes poorly for the future of small cetaceans and for other marine megafauna taxa, which face even greater and more complex conservation challenges.

Fisheries are widely considered the primary threat to small cetaceans^11,17–19^. While industrial fisheries are known to catch large numbers of small cetaceans every year^17,20^, here we provide the first quantitative evidence that it is in fact the small-scale fisheries which have the largest impact on extinction risk. While the number of small cetacean catches in small-scale fisheries is largely unknown^19^, these fisheries are intensively focused in shallow waters where they overlap with those species which have smaller and more fragmented populations^19^ and are therefore less resilient to fisheries mortalities. The threat from small-scale fisheries is further compounded by their abundance in low- and middle-income nations, where these fisheries are most critical for food security and where the capacity for management intervention is most restricted^19,30,31^. Similarly, the degradation of marine habitats—primarily in industrialised regions like Europe, North America, and Central and South-East Asia—places additional pressure on small cetacean populations through loss of suitable habitats and pollutant impacts on their health and reproductive status^21,32^. Despite fisheries and habitat degradation likely being the main drivers of extinction risk in small cetaceans, we find research efforts on these threats, and especially on small-scale fisheries, to be vastly underrepresented in the scientific literature. If researchers, conservationists, and managers are serious about halting the decline of small cetaceans then there needs to be a major shift in the prioritisation of research focus and research funding within the community towards priority threats.

Ongoing programmes such as the International Whaling Commission’s Bycatch Mitigation Initiative and the IUCN’s Integrated Conservation Planning for Cetaceans, which make specific priorities of assessing and tackling small-scale fisheries bycatch, suggest that a re-prioritisation in research efforts may well be underway. Whilst continued documentation of threats and their impacts is critical in motivating change, there is a particular need to focus on solution-oriented research. Presently, there is still relatively little research on the human dimension of threats to small cetaceans (e.g. sociology, economics, management, and governance), but it is these human dimensions which will ultimately underpin the success or failure of conservation efforts.

A shift in research effort alone will be ineffectual without the meaningful and appropriate transfer and application of findings into the management of small cetaceans. For transboundary species, like many small cetaceans, multilateral cooperation is needed for effective management. Unlike their chondrichthyan counterparts, whose protections are often disjointed and irregular^10,33^, marine mammals generally benefit from “joined-up” management regimes across species and geographies. Intentional capture in fisheries are largely illegal worldwide and small cetaceans receive widespread support for their conservation through bodies and agreements such as the International Whaling Commission, the Convention on the Conservation of Migratory Species of Wild Animals (CMS), and the Convention on International Trade in Endangered Species of Wild Fauna and Flora (CITES). In addition, there are a wide variety of approaches available to mitigate the catch of small cetaceans in those countries that have the capacity to implement them^34^. Yet, our analyses showed little evidence of declining extinction risk with increasing fisheries management strength, providing additional evidence to support the increasing criticism of those nations who are capable of but who have failed to make progress in this area^35,36^. Perhaps new, outward facing initiatives like the United States’ new import provisions under the Marine Mammal Protection Act, which promise sanctions for those who fail to protect marine mammals from fisheries and offers economic incentives for those who do, might provide some of the necessary motivation for change. Certainly, a substantial improvement in the application of available management strategies, and research exploring new or improved methods will be critical for the long-term future of small cetaceans.

What is clear is that ongoing failure to reverse, or even simply to halt, the declines of small cetaceans does not bode well if we are serious about tackling marine biodiversity-loss and avoiding an Anthropocene mass extinction^37^. Whilst successful conservation of small cetaceans faces many complex challenges and barriers to success, the taxa are still arguably among the lower hanging fruits of marine conservation. There is generally widespread public support for conservation, consumptive use is rare, there are few ongoing targeted fisheries, and small cetaceans have not experienced the dramatic historical declines seen in some other marine taxa. If we can’t halt the decline of small cetaceans, what are our chances of saving the many marine species heading in the same direction?

## Materials and methods

All analyses were carried out using R v4.1.0 with RStudio v2021.09.0^38,39^ and ArcGIS Pro v3.1.0^40^.

### Threatened status and population trends

To assess and contextualise the threatened status of small cetaceans, we used data from the IUCN Red List^16,28^. The IUCN Red List is the most commonly used method to assess the extinction risk of species at the global scale^41^. Efforts to assess and periodically re-assess species are led and co-ordinated by the various specialist groups of the IUCN Species Survival Commission, alongside expert contributors from around the world. Full details of assessment methodology can be found on the IUCN’s website (http://www.iucnredlist.org) and in associated peer-reviewed publications^42^. We extracted current and historical IUCN Red List categorisation of species and associated population trends for all small cetaceans (*n* = 77), great whales (*n* = 16), pinnipeds (*n* = 36), marine chondrichthyans (*n* = 1,241), and marine ray-finned bony fishes (gigaclass Actinopterygii, *n* = 10,239). IUCN Red List data was extracted for the years 1996–2023, assessments made between 1996 and 2002 use IUCN Categories and Criteria Version 2.3, those made after 2002 use Version 3.1^42^.

IUCN Red List Categories divide species into extinct (Extinct, Extinct in the Wild) and extant. Extant species are further sub-divided into threatened (Vulnerable, Endangered, and Critically Endangered), currently non-threatened (Least Concern, Near Threatened), and those for which there is too little data for assessments to be made (Data Deficient). Species in the higher threatened categories are under sequentially increasing extinction risk. Species are also assigned a population trend status of Increasing, Stable, Decreasing, or Unknown. A Red List Index^16^ was calculated by year across all species considered, whereby Extinct species are assigned a value of 0.0, Critically Endangered a value of 0.2, Endangered 0.4, Vulnerable 0.6, Near Threatened 0.8, and Least Concern 1.0, with the Index calculated as the mean of these values across the species considered.

To provide a base estimate the percentage of small cetaceans, great whales, pinnipeds, chondrichthyans, and ray-finned fishes that are threatened we assume that the relative proportions of threatened and unthreatened groups in Data Deficient species is equal to that of data-sufficient species. This estimate is contrasted against the predicted outputs of our following analyses.

$$Threatend\,\, \left(estimate\,\, \%\right)= \frac{Critically\,\, Endangered+Endangered+Vulnerable}{Total \,\,assessed- Data\,\, Deficient}$$

### Species attributes

The extinction risk of vertebrates is closely linked to biological and geographical attributes across a wide range of taxa^10,12,43–45^. In the marine realm, much of the recent research in this area has focused on bony fish and chondrichthyans. From a biological perspective, maximum size, often used as a proxy for growth rate and time to maturation, and reproductive or population growth rates are key predictors of extinction risk^10,12,46^. Maximum size varies widely even among small cetaceans, ranging from the Vaquita (*Phocoena sinus*) at 1.5 m to the 12.8 m Baird’s Beaked Whale (*Berardius bairdii*). However, unlike bony fish and chondrichthyans, population growth rates of small cetacean are broadly similar across species at 3–8% per year^26,27^ and is therefore unlikely to be a major predictor of varying threatened status. From a geographical perspective, distribution ranges and distribution depths may be linked with extinction risk. For example, in transboundary species like many chondrichthyans, species spread across many countries appear to be at elevated extinction risk, likely a result of disjointed management approaches^10,33^. Those species restricted to shallower depth ranges and closer to shore are also at heightened extinction risk because they are more exposed to anthropogenic impacts, such as fisheries, and have less natural refugia^10–12,28^. Similarly, small cetaceans are largely transboundary and species distributions range from shallow water, coastal obligates to offshore oceanics. For small cetaceans we extracted maximum size from SeaLifeBase (http://www.sealifebase.se) and the number of countries across which each species was distributed from the IUCN Red List (http://www.iucnredlist.org). The maximum water depth of species distribution was extracted from the IUCN Red List, which provides the only standardised global classifications of this type^16^. Where a specific value was not given, species were assigned as Riverine (20 m), Marine Coastal (50 m), Marine Neritic (100 m), Epipelagic (200 m), Mesopelagic (1000 m), or Bathypelagic (4000 m) based on their IUCN Red List Habitat Classification Scheme designation.

### Identifying key threats and defining proxies

Threats to small cetacean species were also extracted from the IUCN Red List. Threats are ranked into six major categories: High Impact, Medium Impact, Low Impact, No/Negligible Impact, Past Impact, and Unknown. To identify the key threat faced by small cetaceans, we extracted only those threats considered to have High or Medium impact (Fig. 2). Once the key threats were identified, a series of proxies for these were compiled for analysis. Threat proxies were compiled for 163 countries with marine waters.

Re-estimated marine fisheries catch from large- (industrial) and small-scale (artisanal and subsistence) fisheries for 2019 were collated from the Seas Around Us database^47^ and were used as proxies for fishing pressure. Fisheries governance strength might be expected to mediate the impact of fisheries on extinction risk. Fisheries Management Index from the Ocean Health Index (http://www.oceanhealthindex.org), which is extrapolated from research into the predictors of relative fisheries management efficacy among countries^31^, were used as a proxy for fisheries management strength. Commercial, passenger, and recreational vessel traffic was extracted from World Bank vessel density maps for 2015–2021^48^ and represented ship-strike risk and noise pollution measures from both sectors. Additionally, the mean number of oil and gas rig counts per country for 2017–2021 (https://rigcount.bakerhughes.com/intl-rig-count) was taken as a measure of the intensity of oil and gas exploration and drilling activity.

FAO crop production, livestock production and forestry production data for 2017–2021 were compiled as a measure of the relative levels of effluent and pollutants associated with agricultural and forestry activity^49,50^. Estimates for untreated wastewater discharge were also compiled^51^; untreated wastewater includes that which originates from agriculture and forestry as well as other sources such as urban, industrial, and military uses. Annual plastic waste flow into the marine environment^52^ was used as a proxy for human and industrial garbage and solid waste inputs.

### Assessing the predictive importance of key threats and species attributes

We used key threat proxies and species attributes (Supplementary Information) to model the IUCN Red List categories of small cetaceans to analyse the relative importance of threats and attributes in predicting extinction risk. IUCN Red List categories were encoded in line with the IUCN Red List Index^16^. The nine Data Deficient small cetacean species were excluded from the analysis. Fisheries Management Index scores for each species were calculated as the mean of the Fisheries Management Index of all countries across which a species is distributed. All other independent variables were summed across all countries in which a species is distributed and standardised by the total coastline length. All independent variables were log-transformed to improve their distributions.

The model was built using eXtreme Gradient Boosting (XGBoost)^53^, which is a form of Boosted Regression Tree. XGBoost is a powerful predictive model able to handle non-linear relationships between the dependent and independent variables and complex variable interactions, and is resilient to co-linearity among independent variables^54^. However, to avoid redundancy within independent variables submitted to modelling, we tested for evidence of high co-linearity (*R* > 0.8) using Pearson’s correlations. Untreated wastewater was highly co-linear with crop agriculture (*R* = 0.95), livestock agriculture (*R* = 0.88), and forestry production (*R* = 0.96). Further, crop agriculture and forestry production were also highly co-linear (*R* = 0.95), as were crop agriculture and livestock agriculture (*R* = 0.87). Untreated wastewater was selected to be retained for analysis because the variable is considered a more holistic proxy for effluent release into coastal environments.

First, the model hyperparameters for eta (the learning rate), maximum tree depth (the complexity of variable interactions), minimum child weight (minimum sum of instance weight per child node), subsample (subsample ratio of the training instance), and gamma (the maximum loss reduction) were tuned using 3-fold cross validation. Both subsample and gamma hyperparameters are used to reduce the likelihood of overfitting. Early stopping was used to tune the number of trees in the model. Monotonic constraints were added to the model to reflect our existing understanding of extinct risk, whereby species living in deeper offshore waters are less exposed to human impacts and therefore at lower extinction risk^11,12,19^ and where threat proxies increase extinction risk should also increase^16^. Monotonic constraints serve to make the model more stable and generalisable. Because of the high imbalance among IUCN Red List Index values (e.g., 3 species with an Index value of 0.2 and 42 species with an index value of 1) we weighted values inversely to their proportional contribution. Root mean squared error was used as the measure of model fit. A total of 1800 unique combinations of hyperparameters were considered. The hyperparameters which produced the best root mean squared error during the tuning stage were eta = 0.2, maximum tree depth = 2, minimum child weight = 1, subsample = 0.7, and gamma = 0.1. The number of trees selected was 69.

The final model was fitted using 10,000 bootstrap iterations, within which data were randomly split into an 80%/20% train/test sets. Monotonic constraints and data weights were retained in the final model. For each bootstrap iteration of the model we extracted bias (average difference between real and predicted IUCN Red List Index values), relative importance of independent variables, the marginal effects for each variable, and we made predictions for Data Deficient species. There was little evidence for bias across the 10,000 bootstrapped iterations (2.49 × 10^−2^ [95% CI 2.41 × 10^−2^ to 2.57 × 10^−2^]). Root mean squared error of the final model was (6.81 × 10^−2^ [95% CI 6.80 × 10^−2^ to 6.82 × 10^−2^]).

### Do small- or large-scale fisheries have the larger causal impact on extinction risk

To explore the relationship between fisheries and extinction risk we assessed and subsequently compared the causal effects of small- and large-scale fisheries. To examine causality, rather than just predictive power, we applied a structured causal model framework using Directed Acyclic Graphs with a backdoor criterion. We proposed a causal structure for the effect of fisheries on extinction risk whereby fisheries management controls fishing pressure, but not bycatch risk because few bycatch mitigation programs are implemented globally^35,36^, and traits which influence fisheries exposure (maximum depth and number of countries a species is distributed across) moderate the causal impact of fisheries (Fig. 5). Causal effects on extinction risk (IUCN Index) were then modelled using two separate generalized linear models for small- and large-scale fisheries independently. The effects of both fisheries types could not be modelled together because they are strongly co-linear (*r* = 0.900). Models included fisheries pressure (Sea Around Us catches); the interaction of fishing pressure and maximum depth and fishing pressure and number of countries (representing the moderating effect of these variables); and fisheries management (Fisheries Management Index) as a control variable. Causal models for other key threats were considered but required more complex model structures and could not be fit because of the low sample size (number of species) available.

### Does research effort reflect the relative importance of threats?

The relative level of research effort in each threat was derived from the peer-reviewed literature. Searches were carried out in Web of Science (http://www.webofscience.com). All searches were conducted on the 5th of March 2024. Search results were restricted to include only articles written in English that were published between 1900 and the date on which searches were conducted. Searches were constrained to title and abstract content only and limited to only peer-reviewed publications. A series of initial search strings using Boolean logic were explored, combining terms relating to small cetaceans and seeking to maximise the number of relevant search returns whilst minimising the inclusion of extraneous results. The chosen search string combined the terms “odontocet*”, “toothed whale”, “dolphin”, “porpoise”, “beaked whale”, and the species names of all 77 species explored in this study. Subsequent searches were then run to identify literature relevant to each specific threat, by appending additional parameters to the initial search. Full details of the Boolean logic search strings used are available (Supplementary Information).

### Supplementary Information

Supplementary Information 1.

Supplementary Information 2.

## Data Availability

All data are freely available in the main text, supplementary information, or from their original source. All code used in the analysis is made available upon request to the corresponding author.
