# Supplementary material for: Management and research efforts are failing dolphins, porpoises, and other toothed whales
Source: Sci Rep. 2024 Mar 25;14:7077. doi: 10.1038/s41598-024-57811-7 (PMC10963780; doi:10.1038/s41598-024-57811-7)
Supplement: Supplementary file 2 — Supplementary Information 2. [file 41598_2024_57811_MOESM2_ESM.docx]

Supplementary Material

Management and research efforts are failing dolphins, porpoises and other toothed whales

Andrew J. Temple^1*^, Ute Langner^1^, Michael L. Berumen^1^

^1^Red Sea Research Center, King Abdullah University of Science and Technology, Thuwal, Saudi Arabia.

^*^Corresponding author. E-mail: andrew.j.l.temple@gmail.com or andrew.temple@kaust.edu.sa

Full Literature Search

Title or Abstract; Article only

Date of search: 17 March 2024

(“odontocet*” OR “toothed whale” OR “dolphin” OR “porpoise” OR “beaked whale” OR “Cephalorhynchus commersonii” OR “Cephalorhynchus eutropia” OR “Cephalorhynchus heavisidii” OR “Cephalorhynchus hectori” OR “Delphinus delphis” OR “Feresa attenuata” OR “Globicephala melas” OR “Globicephala macrorhynchus” OR “Grampus griseus” OR “Lagenodelphis hosei” OR “Lagenorhynchus acutus” OR “Lagenorhynchus albirostris” OR “Lagenorhynchus australis” OR “Lagenorhynchus cruciger” OR “Lagenorhynchus obliquidens” OR “Lagenorhynchus obscurus” OR “Lissodelphis borealis” OR “Lissodelphis peronii” OR “Orcaella brevirostris” OR “Orcaella heinsohni” OR “Orcinus orca” OR “Peponocephala electra” OR “Pseudorca crassidens” OR “Sotalia guianensis” OR “Sousa chinensis” OR “Sousa plumbea” OR “Sousa sahulensis” OR “Sousa teuszii” OR “Stenella attenuata” OR “Stenella clymene” OR “Stenella coeruleoalba” OR “Stenella frontalis” OR “Stenella longirostris” OR “Steno bredanensis” OR “Tursiops aduncus” OR “Tursiops truncatus” OR “Kogia breviceps” OR “Kogia sima” OR “Delphinapterus leucas” OR “Monodon monoceros” OR “Neophocaena asiaeorientalis” OR “Neophocaena phocaenoides” OR “Phocoena dioptrica” OR “Phocoena phocoena” OR “Phocoena sinus” OR “Phocoena spinipinnis” OR “Phocoenoides dalli” OR “Pontoporia blainvillei” OR “Berardius arnuxii” OR “Berardius bairdii” OR “Berardius minimus” OR “Hyperoodon ampullatus” OR “Hyperoodon planifrons” OR “Indopacetus pacificus” OR “Mesoplodon bidens” OR “Mesoplodon bowdoini” OR “Mesoplodon carlhubbsi” OR “Mesoplodon densirostris” OR “Mesoplodon europaeus” OR “Mesoplodon ginkgodens” OR “Mesoplodon grayi” OR “Mesoplodon hectori” OR “Mesoplodon hotaula” OR “Mesoplodon layardii” OR “Mesoplodon mirus” OR “Mesoplodon perrini” OR “Mesoplodon peruvianus” OR “Mesoplodon stejnegeri” OR “Mesoplodon traversii” OR “Tasmacetus shepherdi” OR “Ziphius cavirostris” OR “Mesoplodon eueu” OR “Inia geoffrensis” OR “Lipotes vexillifer” OR “Platanista gangetica” OR “Platanista minor” OR “Sotalia fluviatilis”)

Search output – 14,363 results.

Remove non-journal articles – 13,633 results.

Remove duplicates – 13,539 results.

Remove articles about sperm whales (*Physeter macrocephalus*) – 13,507 results.

---

Fisheries Search

Title or Abstract; Article only

Date of search: 17 March 2024

(“odontocet*” OR “toothed whale” OR “dolphin” OR “porpoise” OR “beaked whale” OR “Cephalorhynchus commersonii” OR “Cephalorhynchus eutropia” OR “Cephalorhynchus heavisidii” OR “Cephalorhynchus hectori” OR “Delphinus delphis” OR “Feresa attenuata” OR “Globicephala melas” OR “Globicephala macrorhynchus” OR “Grampus griseus” OR “Lagenodelphis hosei” OR “Lagenorhynchus acutus” OR “Lagenorhynchus albirostris” OR “Lagenorhynchus australis” OR “Lagenorhynchus cruciger” OR “Lagenorhynchus obliquidens” OR “Lagenorhynchus obscurus” OR “Lissodelphis borealis” OR “Lissodelphis peronii” OR “Orcaella brevirostris” OR “Orcaella heinsohni” OR “Orcinus orca” OR “Peponocephala electra” OR “Pseudorca crassidens” OR “Sotalia guianensis” OR “Sousa chinensis” OR “Sousa plumbea” OR “Sousa sahulensis” OR “Sousa teuszii” OR “Stenella attenuata” OR “Stenella clymene” OR “Stenella coeruleoalba” OR “Stenella frontalis” OR “Stenella longirostris” OR “Steno bredanensis” OR “Tursiops aduncus” OR “Tursiops truncatus” OR “Kogia breviceps” OR “Kogia sima” OR “Delphinapterus leucas” OR “Monodon monoceros” OR “Neophocaena asiaeorientalis” OR “Neophocaena phocaenoides” OR “Phocoena dioptrica” OR “Phocoena phocoena” OR “Phocoena sinus” OR “Phocoena spinipinnis” OR “Phocoenoides dalli” OR “Pontoporia blainvillei” OR “Berardius arnuxii” OR “Berardius bairdii” OR “Berardius minimus” OR “Hyperoodon ampullatus” OR “Hyperoodon planifrons” OR “Indopacetus pacificus” OR “Mesoplodon bidens” OR “Mesoplodon bowdoini” OR “Mesoplodon carlhubbsi” OR “Mesoplodon densirostris” OR “Mesoplodon europaeus” OR “Mesoplodon ginkgodens” OR “Mesoplodon grayi” OR “Mesoplodon hectori” OR “Mesoplodon hotaula” OR “Mesoplodon layardii” OR “Mesoplodon mirus” OR “Mesoplodon perrini” OR “Mesoplodon peruvianus” OR “Mesoplodon stejnegeri” OR “Mesoplodon traversii” OR “Tasmacetus shepherdi” OR “Ziphius cavirostris” OR “Mesoplodon eueu” OR “Inia geoffrensis” OR “Lipotes vexillifer” OR “Platanista gangetica” OR “Platanista minor” OR “Sotalia fluviatilis”)

**AND** (“Fisheries” OR “Fishing” OR “Bycatch” OR “By-catch”)

Search output – 1,257 results.

Remove non-journal articles – 1,212 results.

Remove duplicates – 1,212 results.

Remove articles about sperm whales (*Physeter macrocephalus*) – 1,212 results.

---

Habitat degradation search

Title or Abstract; Article only

Date of search: 17 March 2024

(“odontocet*” OR “toothed whale” OR “dolphin” OR “porpoise” OR “beaked whale” OR “Cephalorhynchus commersonii” OR “Cephalorhynchus eutropia” OR “Cephalorhynchus heavisidii” OR “Cephalorhynchus hectori” OR “Delphinus delphis” OR “Feresa attenuata” OR “Globicephala melas” OR “Globicephala macrorhynchus” OR “Grampus griseus” OR “Lagenodelphis hosei” OR “Lagenorhynchus acutus” OR “Lagenorhynchus albirostris” OR “Lagenorhynchus australis” OR “Lagenorhynchus cruciger” OR “Lagenorhynchus obliquidens” OR “Lagenorhynchus obscurus” OR “Lissodelphis borealis” OR “Lissodelphis peronii” OR “Orcaella brevirostris” OR “Orcaella heinsohni” OR “Orcinus orca” OR “Peponocephala electra” OR “Pseudorca crassidens” OR “Sotalia guianensis” OR “Sousa chinensis” OR “Sousa plumbea” OR “Sousa sahulensis” OR “Sousa teuszii” OR “Stenella attenuata” OR “Stenella clymene” OR “Stenella coeruleoalba” OR “Stenella frontalis” OR “Stenella longirostris” OR “Steno bredanensis” OR “Tursiops aduncus” OR “Tursiops truncatus” OR “Kogia breviceps” OR “Kogia sima” OR “Delphinapterus leucas” OR “Monodon monoceros” OR “Neophocaena asiaeorientalis” OR “Neophocaena phocaenoides” OR “Phocoena dioptrica” OR “Phocoena phocoena” OR “Phocoena sinus” OR “Phocoena spinipinnis” OR “Phocoenoides dalli” OR “Pontoporia blainvillei” OR “Berardius arnuxii” OR “Berardius bairdii” OR “Berardius minimus” OR “Hyperoodon ampullatus” OR “Hyperoodon planifrons” OR “Indopacetus pacificus” OR “Mesoplodon bidens” OR “Mesoplodon bowdoini” OR “Mesoplodon carlhubbsi” OR “Mesoplodon densirostris” OR “Mesoplodon europaeus” OR “Mesoplodon ginkgodens” OR “Mesoplodon grayi” OR “Mesoplodon hectori” OR “Mesoplodon hotaula” OR “Mesoplodon layardii” OR “Mesoplodon mirus” OR “Mesoplodon perrini” OR “Mesoplodon peruvianus” OR “Mesoplodon stejnegeri” OR “Mesoplodon traversii” OR “Tasmacetus shepherdi” OR “Ziphius cavirostris” OR “Mesoplodon eueu” OR “Inia geoffrensis” OR “Lipotes vexillifer” OR “Platanista gangetica” OR “Platanista minor” OR “Sotalia fluviatilis”)

**AND** (“Agricul*” OR “Forestry” OR “Run-off” OR “Run Off” OR “Pesticide*” OR “Pollut*” OR “Eutrophic*” OR “Oligotrophic” OR “Algal Bloom” OR “Algae Bloom” OR “Effluent” OR “Plastic” OR “Plastics” OR “Microplastic” OR “Microplastics” OR “Garbage” OR “Human Waste” OR “Habitat Loss” OR “Habitat Degradation” OR “Habitat Destruction”)

**NOT** (“Noise” OR “Sound” OR “Sonar”)

Search output – 646 results.

Remove non-journal articles – 620 results.

Remove duplicates – 620 results.

Remove articles about sperm whales (*Physeter macrocephalus*) – 617 results.

---

Other human use of the sea Search

Title or Abstract; Article only

Date of search: 17 March 2024

(“odontocet*” OR “toothed whale” OR “dolphin” OR “porpoise” OR “beaked whale” OR “Cephalorhynchus commersonii” OR “Cephalorhynchus eutropia” OR “Cephalorhynchus heavisidii” OR “Cephalorhynchus hectori” OR “Delphinus delphis” OR “Feresa attenuata” OR “Globicephala melas” OR “Globicephala macrorhynchus” OR “Grampus griseus” OR “Lagenodelphis hosei” OR “Lagenorhynchus acutus” OR “Lagenorhynchus albirostris” OR “Lagenorhynchus australis” OR “Lagenorhynchus cruciger” OR “Lagenorhynchus obliquidens” OR “Lagenorhynchus obscurus” OR “Lissodelphis borealis” OR “Lissodelphis peronii” OR “Orcaella brevirostris” OR “Orcaella heinsohni” OR “Orcinus orca” OR “Peponocephala electra” OR “Pseudorca crassidens” OR “Sotalia guianensis” OR “Sousa chinensis” OR “Sousa plumbea” OR “Sousa sahulensis” OR “Sousa teuszii” OR “Stenella attenuata” OR “Stenella clymene” OR “Stenella coeruleoalba” OR “Stenella frontalis” OR “Stenella longirostris” OR “Steno bredanensis” OR “Tursiops aduncus” OR “Tursiops truncatus” OR “Kogia breviceps” OR “Kogia sima” OR “Delphinapterus leucas” OR “Monodon monoceros” OR “Neophocaena asiaeorientalis” OR “Neophocaena phocaenoides” OR “Phocoena dioptrica” OR “Phocoena phocoena” OR “Phocoena sinus” OR “Phocoena spinipinnis” OR “Phocoenoides dalli” OR “Pontoporia blainvillei” OR “Berardius arnuxii” OR “Berardius bairdii” OR “Berardius minimus” OR “Hyperoodon ampullatus” OR “Hyperoodon planifrons” OR “Indopacetus pacificus” OR “Mesoplodon bidens” OR “Mesoplodon bowdoini” OR “Mesoplodon carlhubbsi” OR “Mesoplodon densirostris” OR “Mesoplodon europaeus” OR “Mesoplodon ginkgodens” OR “Mesoplodon grayi” OR “Mesoplodon hectori” OR “Mesoplodon hotaula” OR “Mesoplodon layardii” OR “Mesoplodon mirus” OR “Mesoplodon perrini” OR “Mesoplodon peruvianus” OR “Mesoplodon stejnegeri” OR “Mesoplodon traversii” OR “Tasmacetus shepherdi” OR “Ziphius cavirostris” OR “Mesoplodon eueu” OR “Inia geoffrensis” OR “Lipotes vexillifer” OR “Platanista gangetica” OR “Platanista minor” OR “Sotalia fluviatilis”)

**AND** (((“Noise” OR “Sound” OR “Strike” OR “Lane” OR “Sonar”)

**AND** (“Anthropogenic” OR “Under Water” OR “Underwater” OR “Vessel” OR “Traffic” OR “Boat” OR “Ship*”)) OR (“Oil and Gas” or “Oil & Gas” OR “Drill*” OR “Pile” OR “Oil Rig” OR “Oil Platform”) OR (“Offshore Construction” OR “Wind Farm” OR “Wind Turbine” OR “Tidal Turbine”))

Search output – 682 results.

Remove non-journal articles – 572 results.

Remove duplicates – 572 results.

Remove articles about sperm whales (Physeter macrocephalus) – 569 results.

---

Small-scale fisheries Search

Title or Abstract; Article only

Date of search: 17 March 2024

(“odontocet*” OR “toothed whale” OR “dolphin” OR “porpoise” OR “beaked whale” OR “Cephalorhynchus commersonii” OR “Cephalorhynchus eutropia” OR “Cephalorhynchus heavisidii” OR “Cephalorhynchus hectori” OR “Delphinus delphis” OR “Feresa attenuata” OR “Globicephala melas” OR “Globicephala macrorhynchus” OR “Grampus griseus” OR “Lagenodelphis hosei” OR “Lagenorhynchus acutus” OR “Lagenorhynchus albirostris” OR “Lagenorhynchus australis” OR “Lagenorhynchus cruciger” OR “Lagenorhynchus obliquidens” OR “Lagenorhynchus obscurus” OR “Lissodelphis borealis” OR “Lissodelphis peronii” OR “Orcaella brevirostris” OR “Orcaella heinsohni” OR “Orcinus orca” OR “Peponocephala electra” OR “Pseudorca crassidens” OR “Sotalia guianensis” OR “Sousa chinensis” OR “Sousa plumbea” OR “Sousa sahulensis” OR “Sousa teuszii” OR “Stenella attenuata” OR “Stenella clymene” OR “Stenella coeruleoalba” OR “Stenella frontalis” OR “Stenella longirostris” OR “Steno bredanensis” OR “Tursiops aduncus” OR “Tursiops truncatus” OR “Kogia breviceps” OR “Kogia sima” OR “Delphinapterus leucas” OR “Monodon monoceros” OR “Neophocaena asiaeorientalis” OR “Neophocaena phocaenoides” OR “Phocoena dioptrica” OR “Phocoena phocoena” OR “Phocoena sinus” OR “Phocoena spinipinnis” OR “Phocoenoides dalli” OR “Pontoporia blainvillei” OR “Berardius arnuxii” OR “Berardius bairdii” OR “Berardius minimus” OR “Hyperoodon ampullatus” OR “Hyperoodon planifrons” OR “Indopacetus pacificus” OR “Mesoplodon bidens” OR “Mesoplodon bowdoini” OR “Mesoplodon carlhubbsi” OR “Mesoplodon densirostris” OR “Mesoplodon europaeus” OR “Mesoplodon ginkgodens” OR “Mesoplodon grayi” OR “Mesoplodon hectori” OR “Mesoplodon hotaula” OR “Mesoplodon layardii” OR “Mesoplodon mirus” OR “Mesoplodon perrini” OR “Mesoplodon peruvianus” OR “Mesoplodon stejnegeri” OR “Mesoplodon traversii” OR “Tasmacetus shepherdi” OR “Ziphius cavirostris” OR “Mesoplodon eueu” OR “Inia geoffrensis” OR “Lipotes vexillifer” OR “Platanista gangetica” OR “Platanista minor” OR “Sotalia fluviatilis”)

**AND** (“Fisheries” OR “Fishing” OR “Bycatch” OR “By-catch”)

**AND** (“Small Scale” OR “Artisanal” OR “Subsistence”)

Search output – 131 results.

Remove non-journal articles – 127 results.

Remove duplicates – 127 results.

Remove articles about sperm whales (*Physeter macrocephalus*) – 127 results.
